# Spatial transcriptomics reveals neuroimmune mechanisms of Aβ clearance in immunized Alzheimer’s disease patients

**DOI:** 10.1002/alz.088081

**Published:** 2025-01-03

**Authors:** Lynn van Olst, Brooke Simonton, Alex J Edwards, Anne V Forsyth, Pouya Jamshidi, Mengwei Li, Nate Shepard, Talia Krainc, Benney Argue, Ziyang Zhang, Hang Xu, Jeanette Norman, Rudolph J Castellani, Thomas J Watson, Jinmiao Chen, James AR Nicoll, Delphine Boche, David Gate

**Affiliations:** ^1^ Northwestern University, Chicago, IL USA; ^2^ Singapore Immunology Network (SIgN), Singapore, Singapore Singapore; ^3^ University of Southampton, Southampton United Kingdom; ^4^ Northwestern University Feinberg School of Medicine, Chicago, IL USA; ^5^ National University of Singapore, Singapore, Singapore Singapore; ^6^ University Hospital Southampton NHS Trust, Southampton United Kingdom

## Abstract

**Background:**

Recent advances in Alzheimer’s disease (AD) therapeutics involve immunization against amyloid‐β (Aβ). Post‐mortem brain analysis from the first active Aβ immunotherapy trial indicated clearance of Aβ in some AD patients. Yet, the mechanisms regulating Aβ clearance following immunization remain unknown.

**Method:**

Here, we utilized a novel spatial proteogenomics approach to study brain tissues from 13 AD patients immunized with Aβ. We compared these actively immunized patient brains to tissues from non‐immunized AD patients and non‐neurologic disease controls. Additionally, we used spatial proteogenomics and single‐cell RNA sequencing technologies to investigate the effects of lecanemab, a passive anti‐Aβ drug.

**Result:**

We reveal the transcriptomic neuroimmune response in the Aβ plaque microenvironment following anti‐Aβ immunization. This response is characterized by an increase in genes associated with the TREM2‐APOE axis in microglia of the immunized AD cortex.

**Conclusion:**

Altogether, our data uncover immediate and lasting neuroimmune responses in the AD brain induced by active and passive Aβ vaccination.

